# Supplementary material for: Engraftment of allogeneic iPS cell-derived cartilage organoid in a primate model of articular cartilage defect
Source: Nat Commun. 2023 Feb 20;14:804. doi: 10.1038/s41467-023-36408-0 (PMC9941131; doi:10.1038/s41467-023-36408-0)
Supplement: Supplementary file 1 — Supplementary Information [file 41467_2023_36408_MOESM1_ESM.pdf]

Supplementary Information

Engraftment of allogeneic iPS cell-derived cartilage organoid in a primate model of articular cartilage defect

Kengo Abe, Akihiro Yamashita, Miho Morioka, Nanao Horike, Yoshiaki Takei, Saeko Koyamatsu, Keisuke Okita, Shuichi Matsuda, and Noriyuki Tsumaki

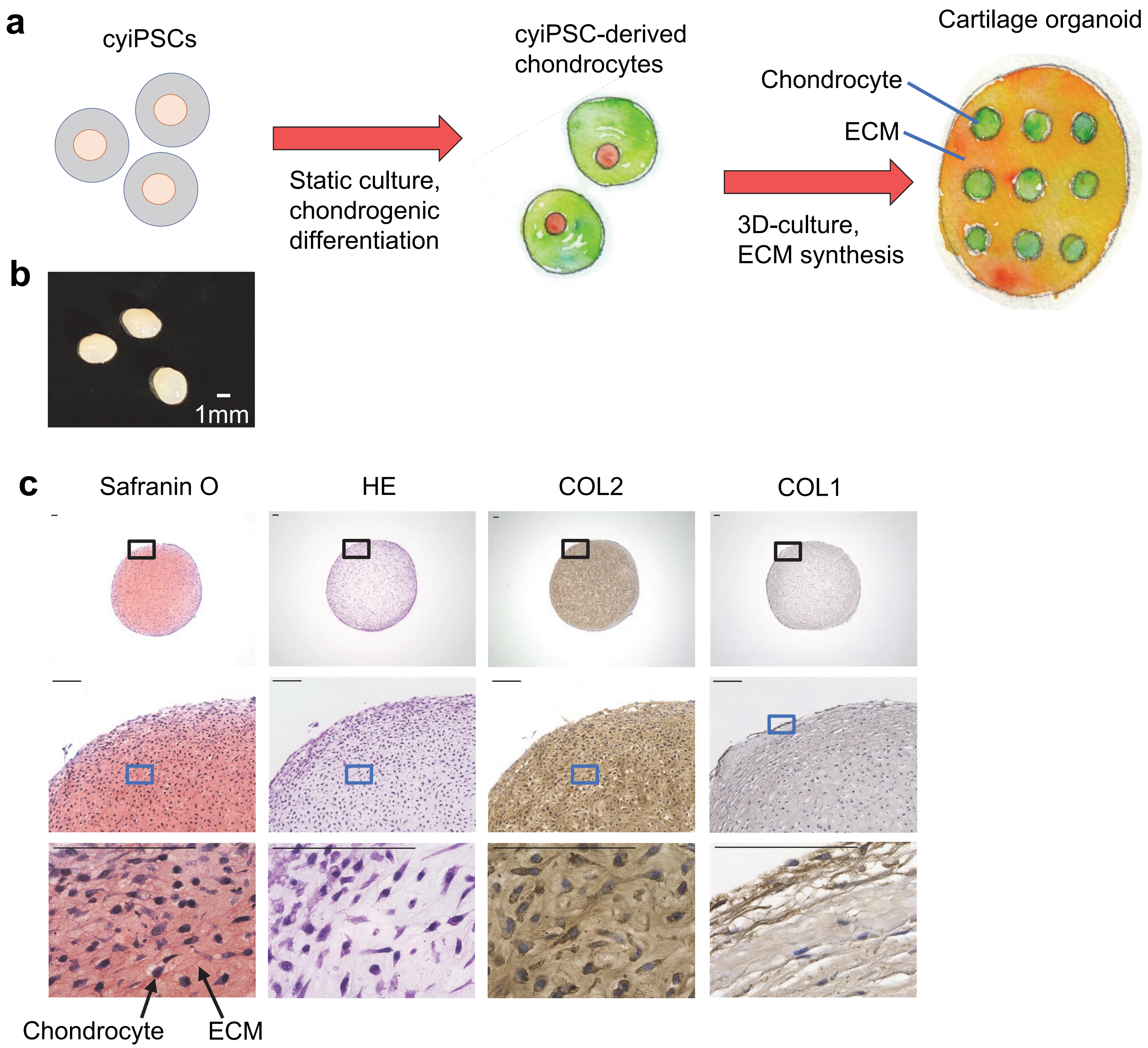

Supplementary Figure 1. cyiPS cell-derived cartilage organoids (cyiPS-Cart)

a) Generation of cyiPS-Cart particle from cyiPSCs.

b) Appearance of a cyiPS-Cart particle. Scale bar, 1 mm.

c) Semi-serial histological sections of cyiPS-Cart particles were stained with safranin O-fast green-iron hematoxylin (safranin O) and hematoxylin-eosin (HE) and immunostained for type II collagen (COL2) and type I collagen (COL1). The black boxed regions in the top row are magnified in the middle row. The blue boxed regions in the middle row are magnified in the bottom row. Scale bars, 100 µm.

Data are representative of three cyiPS-Cart organoids.

Supplementary Figure 2

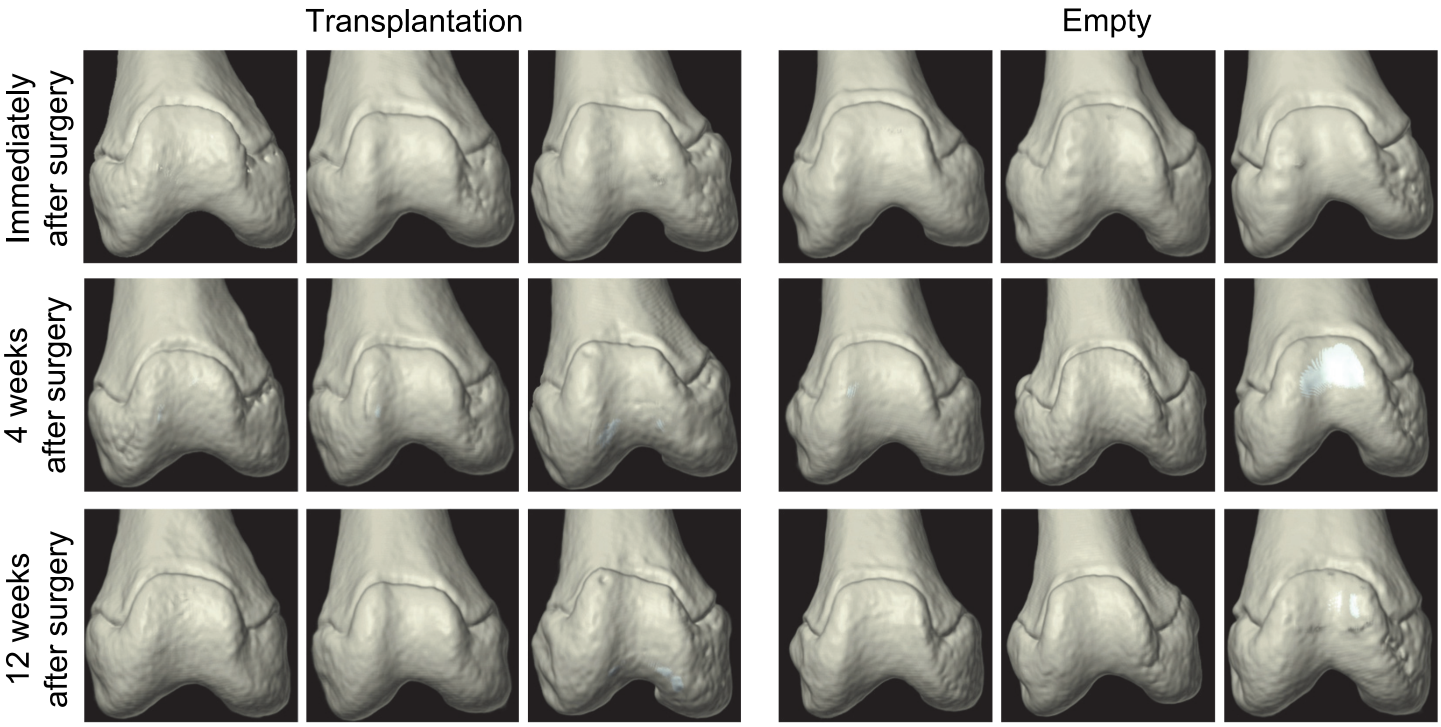

Supplementary Figure 2. Three-dimensional CT images of the knee joints after surgery.
CT was performed on all 12 monkeys immediately and 4 weeks after surgery. Six monkeys were sacrificed 4 weeks after surgery, and CT was performed on remaining monkeys 12 weeks after surgery. Images from the 6 monkeys that were sacrificed 17 weeks after surgery are shown.

Supplementary Figure 3

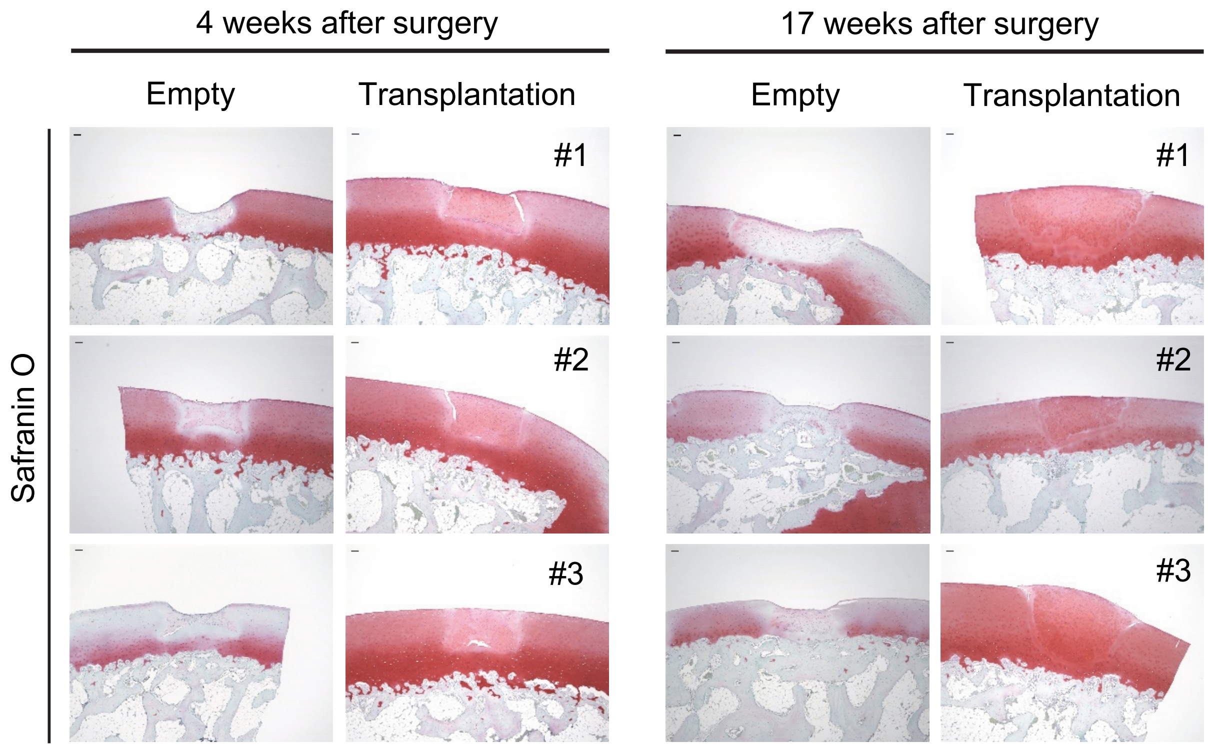

Supplementary Figure 3. Images of histological samples from all 12 monkeys. Each panel represents a different monkey. One representative image for each group is shown in Fig. 3a. Safranin O-fast green-iron hematoxylin staining. Scale bars, 100 µm.

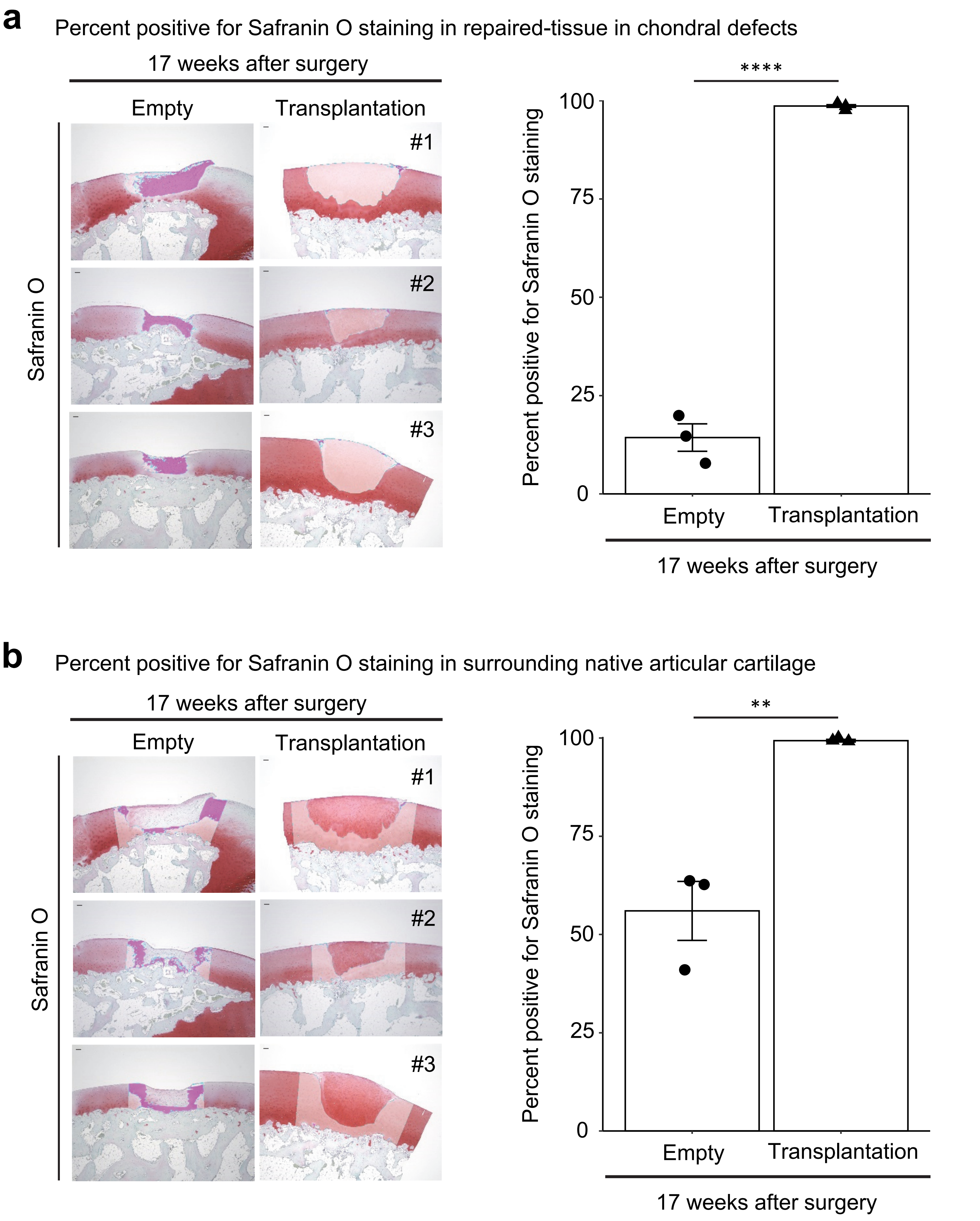

Supplementary Figure 4. Quantitative analysis of safranin O-positive area in repaired tissue in chondral defects and the surrounding native articular cartilage.

a) The safranin O-positive area and total area of the repaired tissues in chondral defects were measured. The safranin O-positive area was divided by the total area.

b) The safranin-O-positive area and the total area of native articular cartilage regions surrounding chondral defects were measured. The safranin O-positive area was divided by the total area.

Scale bars, 100 µm. Error bars denote mean ± SE. ****P < 0.0001, **P = 0.0045 by two-tailed Student's t-test (n = 3 monkeys).

Source data are provided as a Source Data file.

Supplementary Figure 5

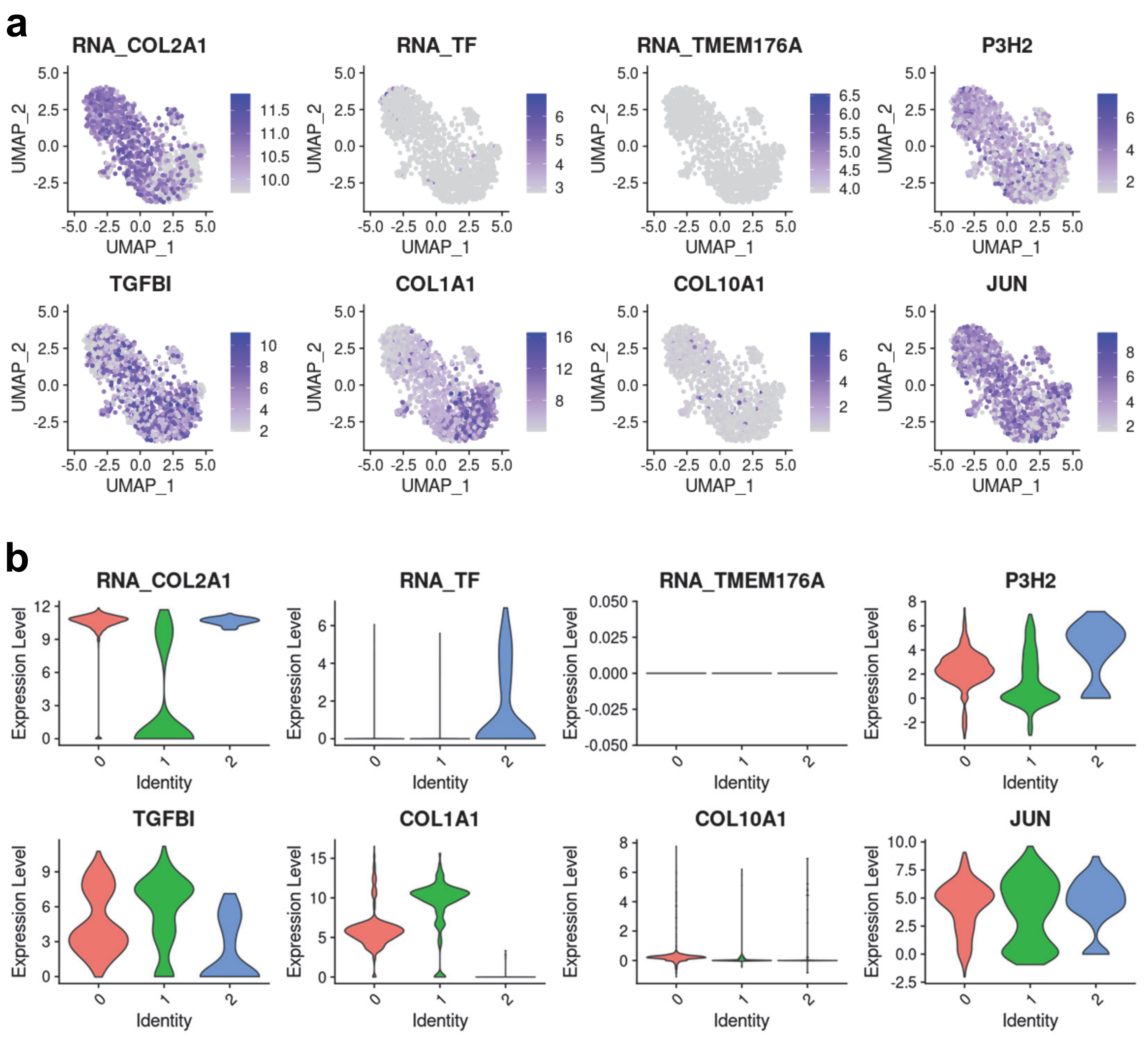

Supplementary Figure 5. scRNA-seq analysis of cyAC, cyFT, pre-transplant cyiPS-Cart, and post-transplant cyiPS-Cart.

a) Expression levels of *COL2A1* and osteoarthritis marker genes are indicated in each cell projected on the UMAP plot using the feature plot function.

b) Expression levels of *COL2A1* and osteoarthritis marker genes are indicated in each cell projected on the UMAP plot using the “VlnPlot” function. The results of Clusters #3 and #4 were omitted because cell numbers in these clusters are few.

Supplementary Figure 6

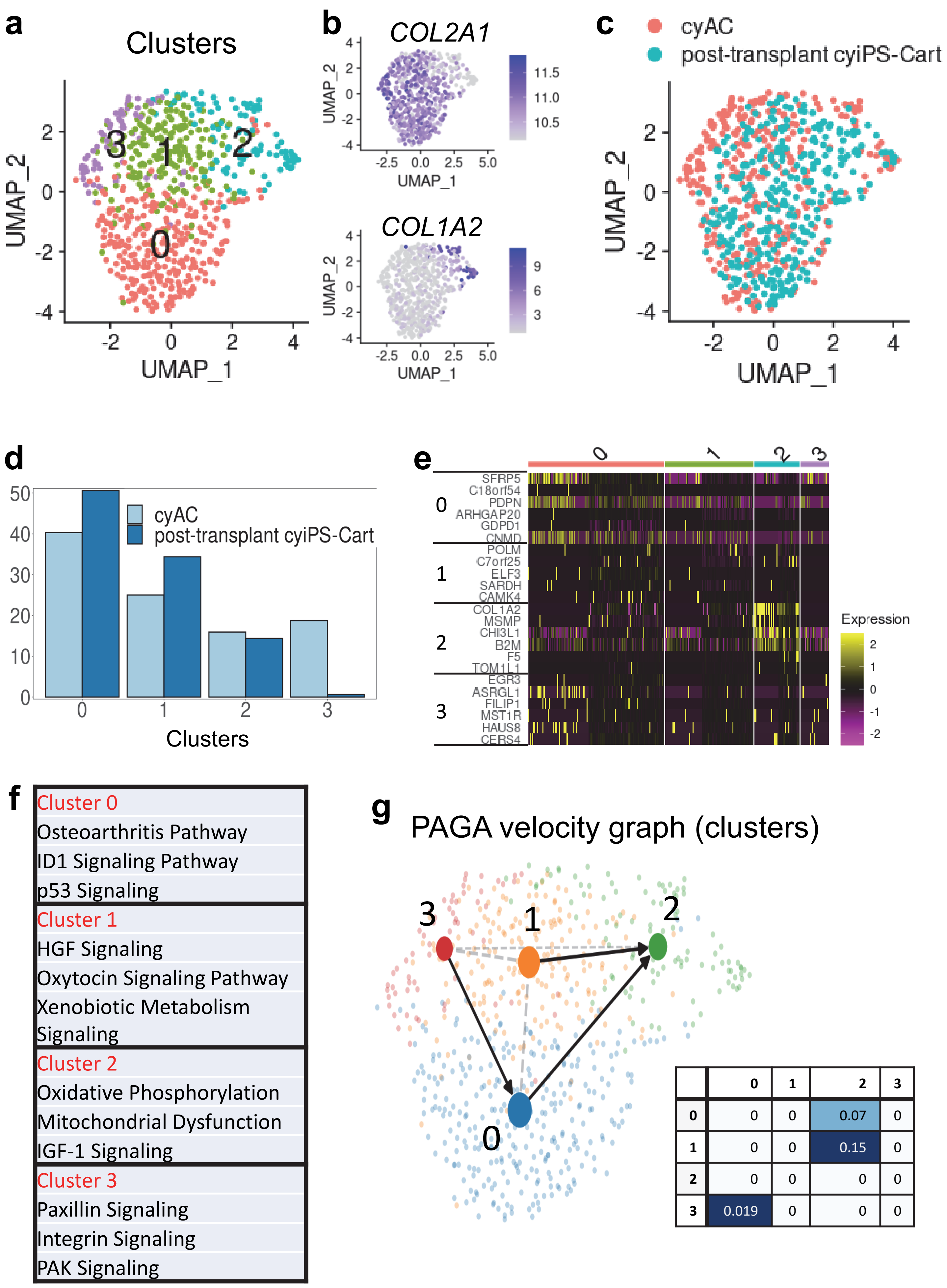

	0	1	2	3
0	0	0	0.07	0
1	0	0	0.15	0
2	0	0	0	0
3	0.019	0	0	0

Supplementary Figure 6. scRNA-seq analysis of cyAC, cyF and post-transplant cyiPS-Cart.

a) After reducing the cell number for each sample to 320, the data from the samples were integrated. The cells were then clustered with a parameter resolution of 0.95 and projected onto the UMAP plots.

b) *COL2A1* and *COL1A2* expression levels are indicated in each cell projected on the UMAP plot using the feature plot function.

c) Distribution of cells in each sample is indicated on the UMAP plot.

d) The ratio of the number of cells in each cell cluster in each sample in (a) is plotted.

e) Heatmap revealing the scaled expression of differentially expressed genes for each cluster defined in (a).

f) Canonical pathways enriched for each cluster based on differentially expressed genes.

g) Post-transplant cyiPS-Cart cells were subjected to RNA velocity analysis using scVelo. The trajectory inference using PAGA was extended by velocity-inferred directionality.

Supplementary Figure 7

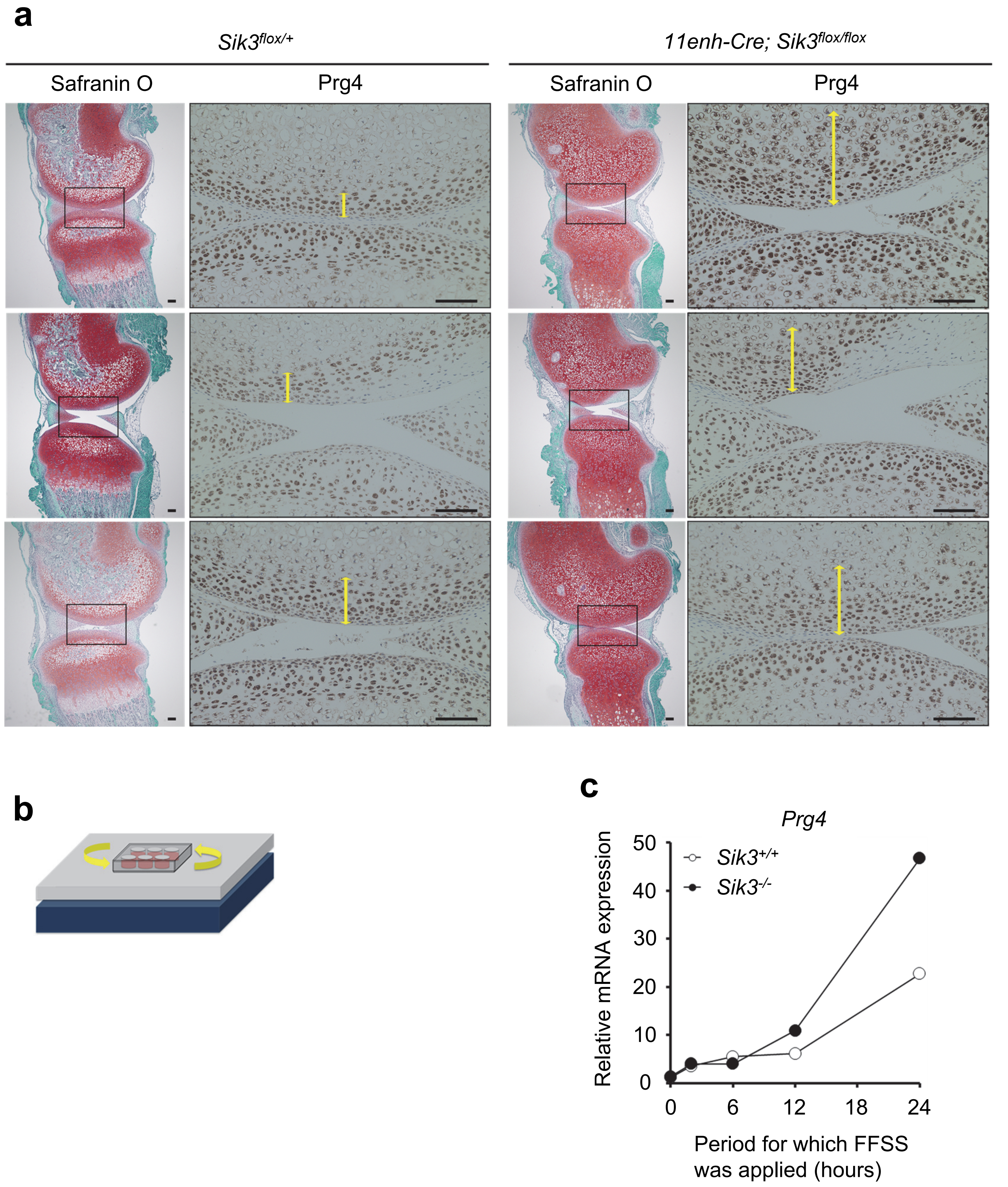

Supplementary Figure 7. Analysis of shear stress and Sik3 contribution to Prg4 expression.

a) Images of histological samples from other three conditional knockout mice and three *Sik3*$^{flox/+}$ mice for Fig. 8d. Scale bars: 100 μm.

b) Schematic representation of the orbital shaker used to subject primary chondrocytes to FFSS.

c) The other data of two independent experiments for Fig. 8e. Source data are provided as a Source Data file.

Supplementary Figure 8

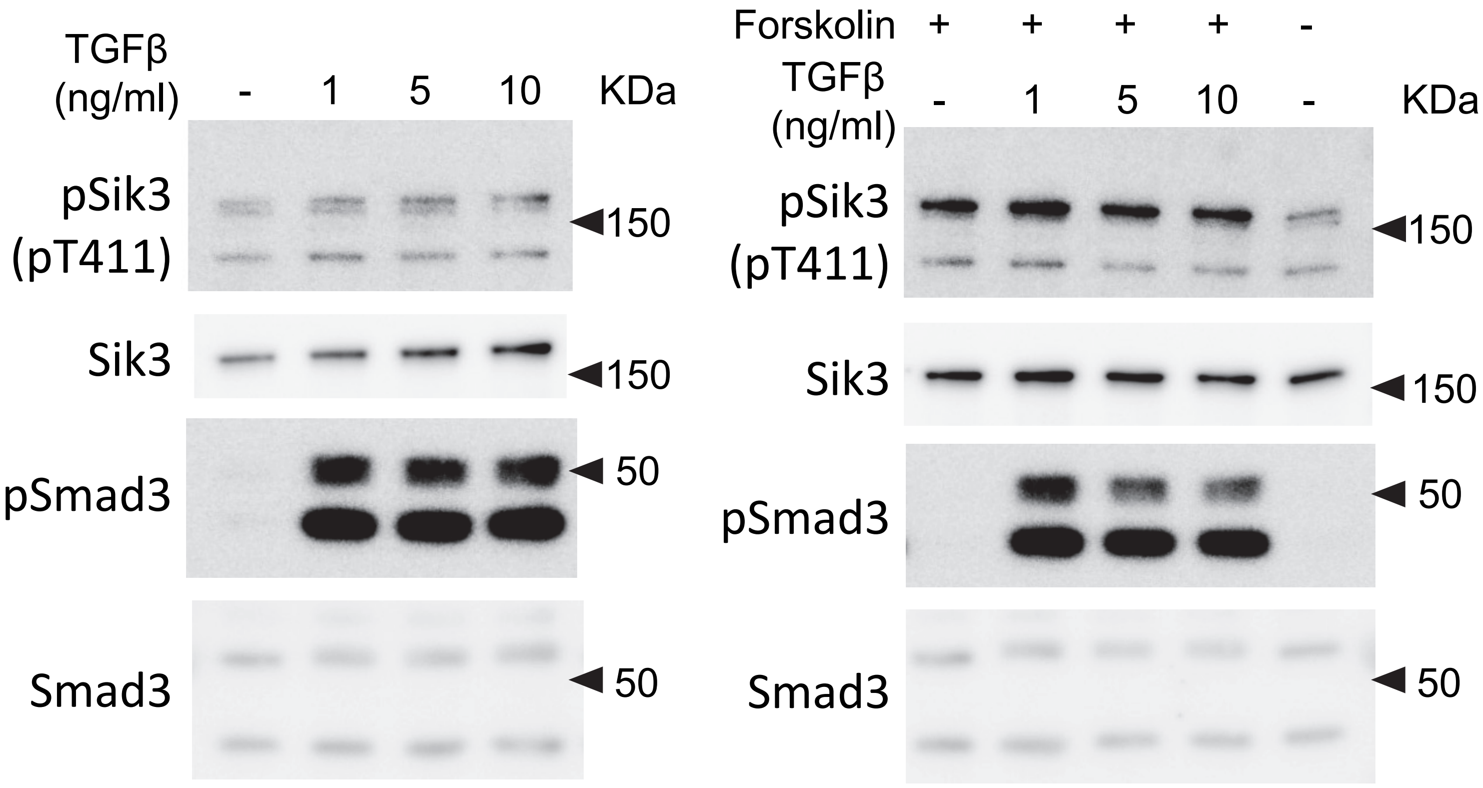

Supplementary Figure 8. Relationship between TGF-β signaling and Sik3 in chondrocytes.

Mouse primary chondrocytes were treated with or without 1, 5 or 10 ng/mL TGF-β1 (*left*), or treated with or without 1, 5 or 10 ng/mL TGF-β1 or 100 μg/mL forskolin (*right*) for 1 h for immunoblot analysis. Data are representative of two independent experiments. Uncropped images are provided in Supplementary Figure 9.

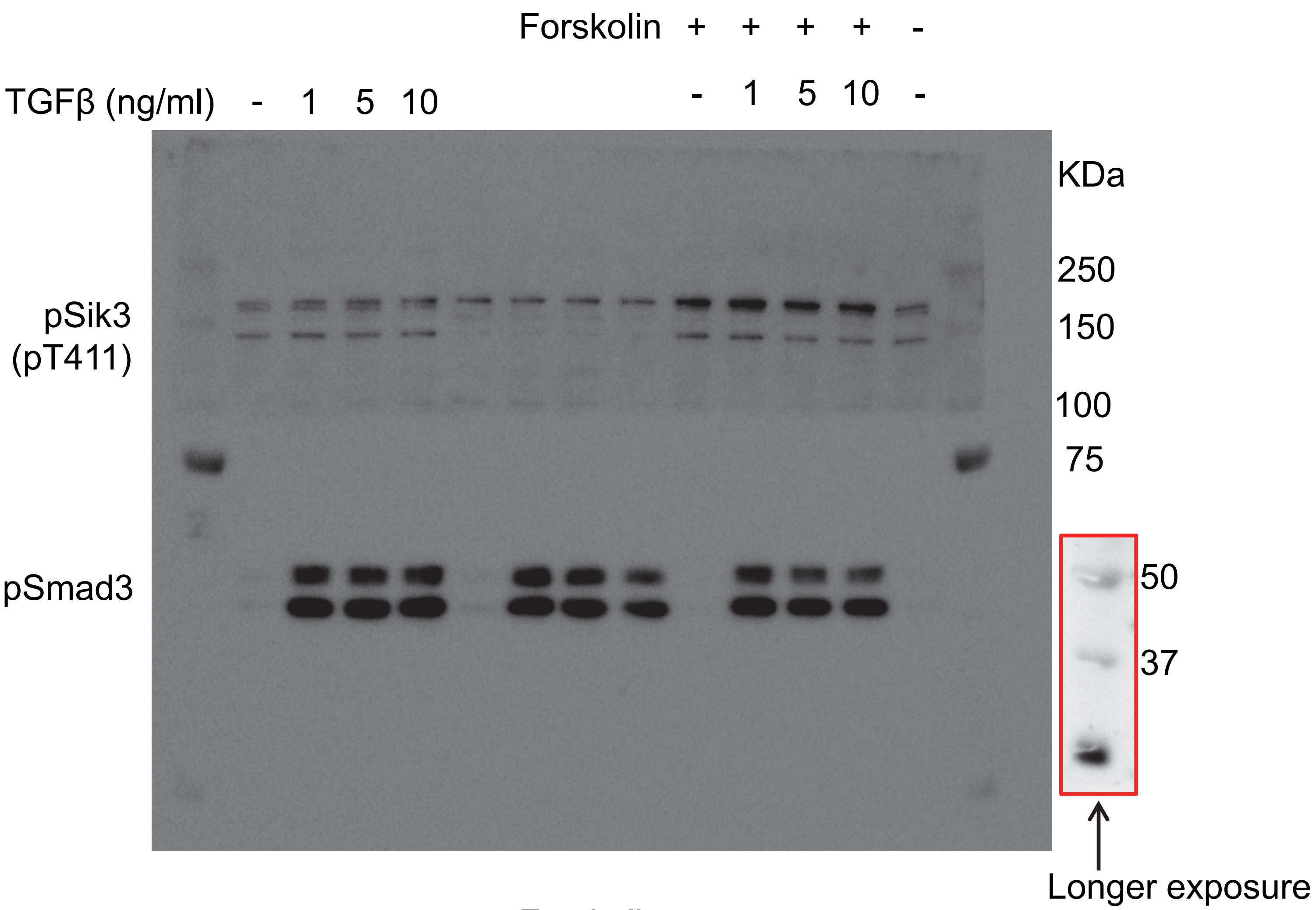

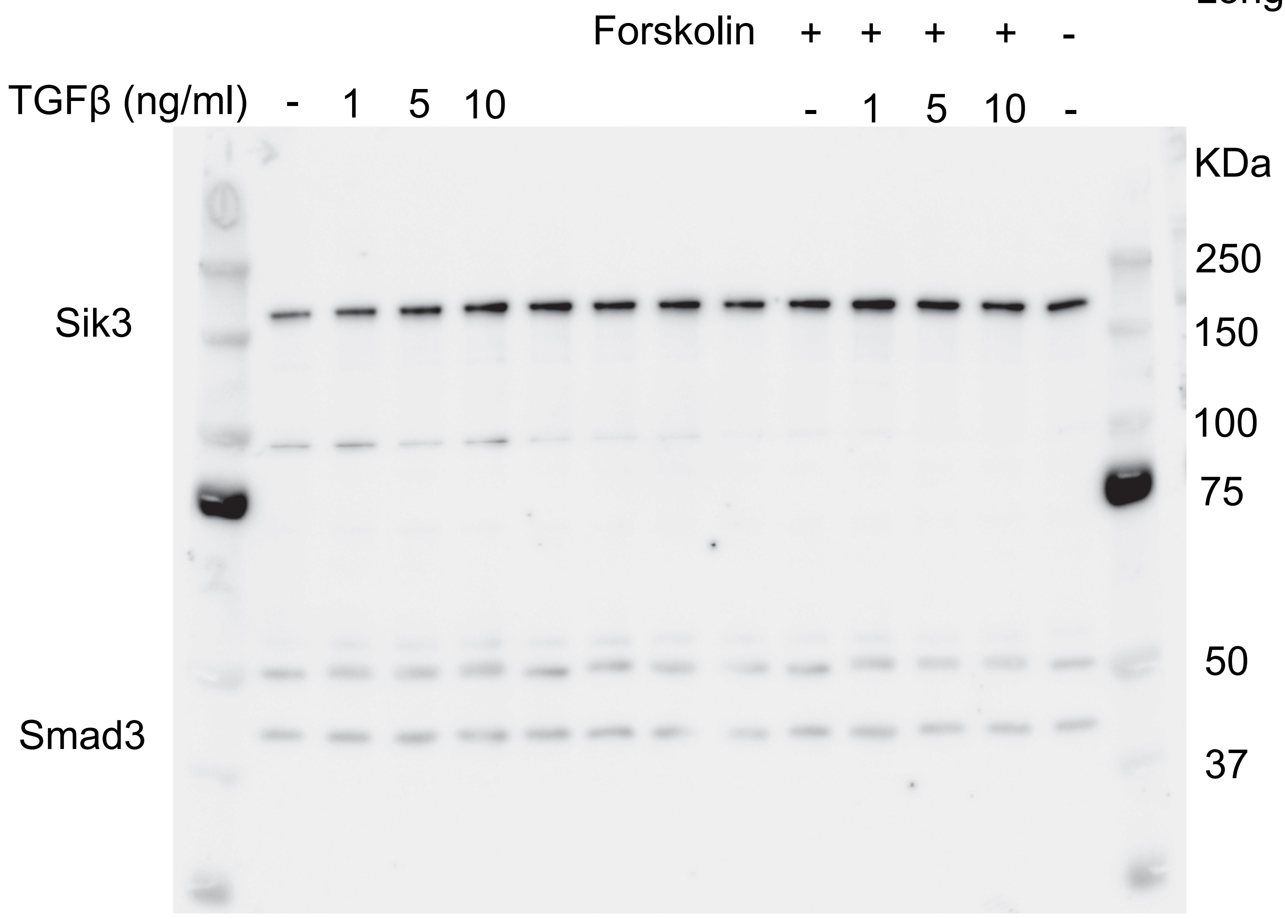

Supplementary Figure 9 Uncropped immunoblot images for Supplementary Fig. 8.

Supplementary Table 1. MHC genotypes

	cyiPSC line (Donor)*	Sacrificed at 4 weeks after surgery			Sacrificed at 17 weeks after surgery		
		#1	#2	#3	#1	#2	#3
**Mafa-F**	F-like4	F-like2	F-like2		F-like2	F-like2<br>F-like4	F-like7
**Mafa-A**	A1*052:02<br>A4*01:04	A1*040:03<br>A1*053:01/02<br>A2*05:21<br>A3*13:16<br>A4*01:07<br>A4*03:02	A1*040:01<br>A1*065:03<br>A3*13:07<br>A3*13:16<br>A4*03:02		A1*022:05<br>A1*040:03<br>A2*05:38 A4*14:15	A1*073:01<br>A1*096:01<br>A2*05:59<br>A4*14:01/08/10<br>A4*14:03/04/13	A1*018:01/09<br>A1*060:04<br>A1*086:01<br>A3*13:02
**Mafa-E**	E-like5<br>E-like11	E-like3<br>E-like6<br>E-like14	E-like1<br>E-like3		E-like1<br>E-like6	E-like3<br>E-like7/10<br>E-like11<br>E-like14	E-like1<br>E-like4
**Mafa-B**	B*095:01<br>B*033:02<br>B*098:10	B*021:01/05/07<br>B*028:03/05/06<br>B*028:04<br>B*051:12<br>B*054:02/03<br>B*060:04/13/19<br>B*060:23<br>B*068:02<br>B*082:05<br>B*105:01<br>B*124:05N<br>B*149:02<br>B*156:01<br>B*205:01	B*050:01/02/06/08/10/11/12<br>B*051:05/08<br>B*060:04/13/19<br>B*065:02/04<br>B*069:05<br>B*072:07<br>B*088:05<br>B*117:03<br>B11L*01:01	n.d.**	B*015:03/08<br>B*036:01/04<br>B*037:01<br>B*045:07<br>B*050:01/02/06/08/10/11/12<br>B*051:04/10/16<br>B*068:06/07/11/13<br>B*079:04<br>B*109:03<br>B*115:05<br>B*167:01N<br>B*180:01/03<br>B*202:01	B*036:01:01/02/04<br>B*037:01<br>B*045:07<br>B*046:12<br>B*050:01/02/06/08/10/11/12<br>B*098:08<br>B*105:01<br>B*149:02<br>B*156:01<br>B*167:01N<br>B*180:01/03	B*007:01/07/09/11<br>B*014:01/02<br>B*034:03<br>B*050:05<br>B*051:05/08<br>B*060:04/13/19<br>B*097:01<br>B*174:01N
**Mafa-I**	I*01:11	I*01:27	I*01		I*01	I*01:27	I*01
**Mafa-DRB**	DRB1*10:07<br>DRB1*03:21	DRB1*03:12/36<br>DRB*W002:05<br>DRB*W020:03<br>DRB*W025:01	DRB1*03:12/36<br>DRB*W021:03<br>DRB*W026:01<br>DRB*W027:03		DRB1*03:03/30<br>DRB1*03:12/36<br>DRB*W001:01<br>DRB*W002:03<br>DRB*W003:02	DRB1*03:03/30<br>DRB1*03:12/36<br>DRB*W001:02<br>DRB*W002:06<br>DRB*W026:01	DRB1*03:12/36<br>DRB*W002:03<br>DRB*W021:01<br>DRB*W027:01:01
**Mafa-DQA1**	DQA1*01:07:01	not done	not done		not done	not done	not done
**Mafa-DQB1**	DQB1*06:08	DQB1*15:03<br>DQB1*18:09	DQB1*06:14<br>DQB1*18:07/26		DQB1*15:01<br>DQB1*17:03/12	DQB1*06:14<br>DQB1*17:03	DQB1*15:01<br>DQB1*17:02
**Mafa-DPA1**	DPA1*02:05	not done	not done		not done	not done	not done
**Mafa-DPB1**	DPB1*15:04	DPB1*05:01<br>DPB1*19:01	DPB1*15:01/13<br>DPB1*19:01		DPB1*03:03/04/05/06<br>DPB1*05:01	DPB1*01:04/08/10/15<br>DPB1*19:06:01	DPB1*03:03/04/05<br>DPB1*05:01

*The cyiPSC line had homozygous MHC haplotype.

**Not determined because the blood sample of this monkey was mistaken.

Supplementary Table 2. Modified Wakitani histological scoring system

Category points	Scores
Cell morphology	
Hyaline cartilage	0
Mostly hyaline cartilage	1
Mostly fibrocartilage	2
Mostly noncartilage	3
noncartilage	4
Matrix staning with safraninO and fast green	
Normal (compared with host adjacent cartilage)	0
Slightly reduced	1
Markedly reduced	2
No metachromatic stain	3
Thickness of cartilage	
>2/3	0
1/3-2/3	1
<1/3	2
Integration of implant with adjacent host cartilage	
Both edges integrated	0
One edge integrated	1
Neither edge integrated	2
Total maximum	11

Supplementary Table 3. List of antibodies used for immunohistochemistry

Antibody	Species	Dilution	Catalog No.	Source
Anti Collagen I	Goat	1:1500	1310-01	Southern Biotech
Anti Collagen II	Goat	1:300	1320-01	Southern Biotech
Anti Lubricin/Proteoglycan 4	Mouse	1:500	MABT400	Milipore
Anti CD3	Rat	1:100	ab11089	abcam
Anti GFP	Rabbit	1:500	NB600-308	NOVUS
Anti mouse immunoglobulin HRP		No dilution	K1497	DAKO
Anti rabbit immunoglobulin HRP	Mouse	1:100	sc-2357	Santa Cruz Biotechnology
Anti goat immunoglobulin HRP	Donkey	1:1000	ab97110	abcam
Alexa Fluor 488 goat anti rat IgG(H+L)	Goat	1:1000	A11006	Invitrogen

Supplementary Table 4. List of primers

Target		F/R	Seqence 5'→3'
monkey	GAPDH	F	CCTTCACACCCTTGCGTATT
monkey	GAPDH	R	TTGATTTTGGAGGGATCTCG
monkey	PRG4	F	GGAGATGTGGGGAAGGGTAT
monkey	PRG4	R	TGCTTTCTTTGCAGATGGTG
mouse	GAPDH	F	TGGATTTGGACGCATTGGTC
mouse	GAPDH	R	TTTGCACTGGTACGTGTTGAT
mouse	ACTB	F	GGCTGTATTCCCCTCCATCG
mouse	ACTB	R	CCAGTTGGTAACAATGCCATGT
mouse	PRG4	F	TGGAGTGCTGTCCTGATTTCAAGAG
mouse	PRG4	R	GGTGATTTGGGTGAGCGTTTGGTA
