# Supplementary material for: Engraftment of allogeneic iPS cell-derived cartilage organoid in a primate model of articular cartilage defect
Source: Nat Commun. 2023 Feb 20;14:804. doi: 10.1038/s41467-023-36408-0 (PMC9941131; doi:10.1038/s41467-023-36408-0)
Supplement: Supplementary file 3 — Description of Additional Supplementary Files [file 41467_2023_36408_MOESM3_ESM.pdf]

File Name: Supplementary Data 1
Description:DEGs between PRG4-positive and -negative cells.

File Name: Supplementary Data 2
Description: IPA on DEGs between PRG4-positive and -negative cells.

File Name: Supplementary Code 1
Description: The scripts that were used for obtaining the results of scRNA-seq analysis (Figs. 5 and 6; Extended Data Figs. 5 and 6).
