# Supplementary material for: Engraftment of allogeneic iPS cell-derived cartilage organoid in a primate model of articular cartilage defect
Source: Nat Commun. 2023 Feb 20;14:804. doi: 10.1038/s41467-023-36408-0 (PMC9941131; doi:10.1038/s41467-023-36408-0)
Supplement: Supplementary file 6 — Reporting Summary [file 41467_2023_36408_MOESM6_ESM.pdf]

nature portfolio

Corresponding author(s): Noriyuki Tsumaki

Last updated by author(s): Jan 6, 2023

Reporting Summary

Nature Portfolio wishes to improve the reproducibility of the work that we publish. This form provides structure for consistency and transparency in reporting. For further information on Nature Portfolio policies, see our Editorial Policies and the Editorial Policy Checklist.

Statistics

For all statistical analyses, confirm that the following items are present in the figure legend, table legend, main text, or Methods section.

n/a	Confirmed	
☐	☒	The exact sample size (*n*) for each experimental group/condition, given as a discrete number and unit of measurement
☐	☒	A statement on whether measurements were taken from distinct samples or whether the same sample was measured repeatedly
☐	☒	The statistical test(s) used AND whether they are one- or two-sided<br>*Only common tests should be described solely by name; describe more complex techniques in the Methods section.*
☒	☐	A description of all covariates tested
☐	☒	A description of any assumptions or corrections, such as tests of normality and adjustment for multiple comparisons
☐	☒	A full description of the statistical parameters including central tendency (e.g. means) or other basic estimates (e.g. regression coefficient) AND variation (e.g. standard deviation) or associated estimates of uncertainty (e.g. confidence intervals)
☐	☒	For null hypothesis testing, the test statistic (e.g. *F*, *t*, *r*) with confidence intervals, effect sizes, degrees of freedom and *P* value noted<br>*Give P values as exact values whenever suitable.*
☒	☐	For Bayesian analysis, information on the choice of priors and Markov chain Monte Carlo settings
☒	☐	For hierarchical and complex designs, identification of the appropriate level for tests and full reporting of outcomes
☒	☐	Estimates of effect sizes (e.g. Cohen's *d*, Pearson's *r*), indicating how they were calculated

Our web collection on statistics for biologists contains articles on many of the points above.

Software and code

Policy information about availability of computer code

Data collection	Three-dimensional CT images were constructed using an image processing software (Aquilion TSX-101A/NA; TOSHIBA, Japan).<br>Percent positive for Safranin O staining in tissues are calculated by BZ-X800 analyzer software (Keyence Corp., Japan).<br>The FUSION FX software (Vilber, France) was used for Western blot quantification.
Data analysis	The statistical analyses were performed using JMP® Pro 16.2.0.<br>For scRNA-seq data analysis, the DropletUtils package in R 3.6.3 (https://cran.r-project.org/), the FlowTrans package47 in R 3.6.3., and the mclust package48 in R 3.6.3. were used.<br>Single-cell RNA seq data was analysed using Seurat v4.0.3 in R 4.1.0.<br>We used Ingenuity Pathway Analysis v01-20-04 (IPA, Qiagen) to detect possible upstream regulators of differentially expressed genes (DEGs).

For manuscripts utilizing custom algorithms or software that are central to the research but not yet described in published literature, software must be made available to editors and reviewers. We strongly encourage code deposition in a community repository (e.g. GitHub). See the Nature Portfolio guidelines for submitting code & software for further information.

March 2021

Data

Policy information about availability of data

All manuscripts must include a data availability statement. This statement should provide the following information, where applicable:

- Accession codes, unique identifiers, or web links for publicly available datasets
- A description of any restrictions on data availability
- For clinical datasets or third party data, please ensure that the statement adheres to our policy

All the original data are available upon request from the authors. Our scRNA-seq datasets are available on GEO accession number GSE206120 and reviewer token cnylewoyxbwtnqz.

Human research participants

Policy information about studies involving human research participants and Sex and Gender in Research.

Reporting on sex and gender	Human research participants are not involved in the study.
Population characteristics	n/a
Recruitment	n/a
Ethics oversight	n/a

Note that full information on the approval of the study protocol must also be provided in the manuscript.

Field-specific reporting

Please select the one below that is the best fit for your research. If you are not sure, read the appropriate sections before making your selection.

☒ Life sciences ☐ Behavioural & social sciences ☐ Ecological, evolutionary & environmental sciences

For a reference copy of the document with all sections, see nature.com/documents/nr-reporting-summary-flat.pdf

Life sciences study design

All studies must disclose on these points even when the disclosure is negative.

Sample size	No sample size calculations were performed. The number of monkeys was chosen so that the sample size was three independent replicates, which is the minimum number for a statistical test and contributes to reducing the number of animals used in the experiments. The numbers of samples were sufficient because the mean values were significantly different between the test group and the control group.
Data exclusions	MHC type was not determined in one monkey, because the blood sample was mistaken. This monkey was included for data collection.
Replication	Experiments were replicated 3-6 times and performed independently 2-3 times.<br>We used multiple samples and the sample numbers are presented and all the data is reproduced in all experiments.
Randomization	Monkeys, mice, and cells were randomly divided into two groups (transplanted and empty).
Blinding	Blinding was performed when scoring cartilage repair (Fig. 3b). In other experiments, blinding was not possible because of presence of unique identifiers highlighting groups.

Reporting for specific materials, systems and methods

We require information from authors about some types of materials, experimental systems and methods used in many studies. Here, indicate whether each material, system or method listed is relevant to your study. If you are not sure if a list item applies to your research, read the appropriate section before selecting a response.

Materials & experimental systems

n/a	Involved in the study
☐	☒ Antibodies
☐	☒ Eukaryotic cell lines
☒	☐ Palaeontology and archaeology
☐	☒ Animals and other organisms
☒	☐ Clinical data
☒	☐ Dual use research of concern

Methods

n/a	Involved in the study
☒	☐ ChIP-seq
☒	☐ Flow cytometry
☒	☐ MRI-based neuroimaging

Antibodies

Antibodies used	Antibodies are listed in Extended DataTable 5 and in the Methods section.
Validation	Antibodies are used under validation using adequate negative and positive control samples.<br>We confirmed the staining by using animal samples when possible, or we rely on the stained samples presented in manufacturer's website. For immunohistochemistry, antibodies from Southern Biotech, Milipore, abcam, and NOVUS were tested by immunohistochemistry. For immunoblotting, antibodies from Abcam, KNEXUS, CST, and Cell signaling were tested by immunoblot.

Eukaryotic cell lines

Policy information about cell lines and Sex and Gender in Research

Cell line source(s)	cyiPSC line (1146A1) was generated from peripheral blood mononuclear cells of a male crab-eating monkey with homozygous MHC, and cyiPSC line (1231C1-G) was similarly generated from that of a female crab-eating monkey with homozygous MHC.
Authentication	Not applicable. No cell lines were used.
Mycoplasma contamination	All cells were negative for mycoplasma contamination.
Commonly misidentified lines (See ICLAC register)	Nothing.

Animals and other research organisms

Policy information about studies involving animals; ARRIVE guidelines recommended for reporting animal research, and Sex and Gender in Research

Laboratory animals	3-4 years old male cynomolgus monkeys(Ina Research, Nagano, Japan) were used for this studey.purchased from Ina Research (Nagano, Japan)<br>Sik3-/- mice, Sik3flox/flox mice, 11Ehn-Cre transgenic mice, and Col11a2-hSIK3 transgenic mice are C57BL/6 mice. Mice with both sexes, aged from at birth to 1 year old were used. We mated mice to prepare those that bear specific genotypes.<br>Animal rooms for mice were controlled to provide temperature of around 23? and around 55% of humidity on a 12h light/dark cycle.
Wild animals	The study did not involve wild animals.
Reporting on sex	We used all male monkeys in the study to avoid gender difference bias.
Field-collected samples	The study did not involve field-collected samples.
Ethics oversight	All methods were performed following relevant guidelines and regulations. Experiments using recombinant DNA were approved by the Recombinant DNA Experiments Safety Committees of Kyoto University (No. 180041) and Osaka University (No. 04794). All animal experiments were approved by the Institutional Animal Committees of the Kyoto University (No. 18-101-14 and No. 16-74-17) and Osaka University (No. 03-044-014).

Note that full information on the approval of the study protocol must also be provided in the manuscript.
